# Relationship between behavioral inhibition/activation system and Internet addiction among Chinese college students: The mediating effects of intolerance of uncertainty and self-control and gender differences

**DOI:** 10.3389/fpubh.2022.1047036

**Published:** 2022-12-28

**Authors:** Zhihao Zhang, Yan Lin, Jia Liu, Guangyu Zhang, Xiaowen Hou, Zequan Pan, Bibing Dai

**Affiliations:** ^1^School of Public Administration, Central South University, Changsha, China; ^2^Department of Psychiatry and Psychology, School of Basic Medical Sciences, Tianjin Medical University, Tianjin, China; ^3^Transplantation Center, The Third Xiangya Hospital of Central South University, Changsha, China

**Keywords:** college students, behavioral inhibition/activation system, intolerance of uncertainty, self-control, Internet addiction, gender differences

## Abstract

**Background:**

Internet addiction is a global public health issue among college students that is associated with a range of negative outcomes. Especially the COVID-19 pandemic has forced them to shift most of their studies and life activities from offline to online, leading to a growing problem of Internet dependence and even Internet addiction. Although previous studies have indicated that the Behavioral Inhibition/Activation System (BIS/BAS) have important effects on college students' Internet addiction, the mechanisms underlying these associations and gender differences are still unclear.

**Aims:**

The present study investigated the mediating roles of intolerance of uncertainty and self-control in the association between BIS/BAS and Internet addiction following the Interaction of Person-Affect-Cognition-Execution model. Gender differences in such associations between variables were also tested.

**Method:**

A total of 747 Chinese college students were surveyed by using Young's Diagnostic Questionnaire for Internet Addiction, BIS/BAS Scales, the Intolerance of Uncertainty Scale and the Brief Self-Control Scale.

**Results:**

The results from the structural equation modeling analysis showed that BIS was positively related to Internet addiction and that BAS had a negative association with Internet addiction. Moreover, intolerance of uncertainty and self-control mediated the relationships between BIS/BAS and Internet addiction. Multi-group analysis further revealed that the associations between BAS and Internet addiction and between intolerance of uncertainty and Internet addiction were stronger among the male students than among female students. The relationship between self-control and Internet addiction was greater in the female sample than in the male sample.

**Conclusions:**

These findings extend our understanding of how BIS/BAS influence Internet addiction among college students and suggest that not only should training approaches based on intolerance of uncertainty and self-control be fully considered, but different intervention programs should be focused on gender sensitivity to maximize the intervention effect.

## Introduction

During the past two decades, access to the Internet has become widespread with the rapid development of information technology. The number of Internet users reached approximately 4.95 billion worldwide by 2022 (1), and Internet usage in developing countries increased from 7.7 to 45.3% between 2005 and 2018 (2). According to recent data released by the China Internet Network Information Center (CNNIC), the number of Chinese Internet users had reached 1.051 billion and the Internet penetration rate had reached 74.4% by June 2022 (3). There is no denying that reasonable Internet use is beneficial in many ways, but excessive Internet use may lead to Internet addiction (4). Internet addiction is conceptualized as an inability to control one's use of the Internet, which eventually causes psychological, social, school, and work problems (5). Many empirical studies have shown that Internet addiction is typically linked with a variety of psychological and behavioral problems, such as anxiety, depression, sleep disorders, poor interpersonal relationships, and even a high risk of suicide (6–10). Internet addiction has become a major global public health problem due to its harmful effects (11). To make matters worse, COVID-19 pandemic has exacerbated the problem. Since various prevention strategies have been adopted to prevent the spread of COVID-19, such as social distancing, quarantine and school closures. The way people work and daily lives have changed dramatically, and many activities have shifted from offline to online, making people inseparable from the Internet (12). As a result, many studies have shown higher rates of online activity and higher rates of Internet addiction than in the pre-pandemic period (13, 14). The risk of Internet addiction among teenagers is often mentioned, but college students are another particularly vulnerable age group. Previous studies have demonstrated that the average prevalence of Internet addiction was approximately 7.02% (15), while the prevalence of Internet addiction among Chinese college students was approximately 11.0% (16), which is 1.6 times higher than that of the general population, and the prevalence rates might further increase in the coming years. Therefore, to develop effective prevention and intervention strategies, it is imperative to identify risk factors and underlying mechanisms associated with college students' Internet addiction.

In recent years, the Interaction-Person-Affect-Cognition-Execution (I-PACE) model has become a prominent theoretical framework to explain Internet addiction (17, 18). The model consists of four components, P-A-C-E, where the P-component refers to the individual's core characteristics, such as personality and psychopathological features. The A-component and C-component refer to the person's affective and cognitive responses to external or internal stimuli, respectively, such as cognitive biases and an urge for mood regulation. Finally, the E-component refers to one's executive function, such as inhibitory control and self-control. Brand explained the formation and maintenance of Internet addiction from the perspective of the interaction of personality, affective, cognitive response and execution, and the I-PACE model identifies personality as a predisposition factor that leads to Internet addiction by influencing the individual's affective-cognitive responses and executive function. Therefore, within the framework of the I-PACE model, this study aims to investigate the influence of personality factors (e.g., behavioral inhibition system/behavioral activation system) on Internet addiction and whether adverse cognitive and affective responses (e.g., intolerance of uncertainty) to environmental stressors and executive functioning (e.g., self-control) play a mediating role in the relationship between personality factors (e.g., behavioral inhibition system/behavioral activation system) and Internet addiction, using a sample of Chinese college students. Furthermore, because males and females differ in core characteristics including biopsychological constitution, psychopathological features, personality, and social cognitions, gender may play a moderating role in the relationships between these variables. Thus, this study will also explore the gender differences among the above variables to further enrich the I-PACE model.

### Behavioral inhibition system/behavioral activation system and Internet addiction

Gray's reinforcement sensitivity theory is known as an effective view for understanding and explaining basic human behaviors, particularly regarding addiction. The theory suggests that there are individual differences in sensitivity to stimuli from two basic brain systems that regulate and control human motivation and behavior, namely, the behavioral inhibition system (BIS) and behavioral activation system (BAS) (19). The former is related to stimuli conditioned for punishment or the termination of rewards and is responsible for regulating avoidance behavior to avert threats and penalties. Thus, individuals with high BIS are more likely to experience negative emotions (e.g., anxiety, fear) and exhibit behavioral inhibition (20). The latter is associated with stimuli relevant to rewards or the termination of punishment and is responsible for regulating the acquisition of rewards and achieving goals. Therefore, high BAS-sensitive people are more prone to feeling positive emotions (e.g., hope, wellbeing) and engage in approach behavior to obtain greater advantage in the world (21). Gray's theory provided an essential perspective for understanding and explaining addiction, but previous findings about the associations between BIS/BAS and Internet addiction were inconsistent. For example, in a survey of 519 middle-school students, BIS and BAS activation were both associated with Internet addiction (21). Another study of 197 middle-school students indicated that BAS activation rather than BIS activation is a significant risk factor for predicting the occurrence of Internet addiction (22). However, a recent study divided 754 college students into addiction and non-addiction groups according to questionnaire results and Internet Gaming Disorder diagnostic criteria, and the results showed that BIS not BAS was statistically correlated with scores on the Internet Addiction Questionnaire (23). We speculate that a variety of factors may contribute to the differences in the results. First, different measurement tools examine different theories and constructs, which can have an impact on the degree of association between variables. Second, the performance and development of reward and punishment sensitivity of participants in the different developmental stages may vary with different living conditions and developmental tasks (23, 24). Third, the analysis of the mixed sample ignored the effect of the gender of the participants on the study results. Although there is no definite conclusion as to whether BIS or BAS or both have a significant effect on Internet addiction, many researchers suggest that the relationship between BIS/BAS and Internet addiction is closely related (25, 26). Moreover, since BIS/BAS is considered as relatively stable traits that is difficult to change directly (27), it is necessary to explore the mediating mechanisms of BIS/BAS leading to Internet addiction, which is helpful for providing a theoretical basis for the prevention and intervention for Internet addiction.

### The mediating roles of intolerance of uncertainty and self-control

Intolerance of uncertainty (IU) may play a mediating role in the association between BIS/BAS and Internet addiction (28–31). IU is defined as a cognitive bias that affects how a person perceives, interprets, and responds to uncertain situations (32). On the one hand, BIS/BAS, as a core characteristic with individual differences, may be linked to IU. First, individuals with high BIS levels are likely to exhibit enhanced associative learning and readily learn to avoid aversive situations (33). Second, BIS is relevant to attempting to escape from or avoid novel, threatening or uncertain contexts, which may lead to negative interpretations of ambiguous stimuli in these environments by individuals (34). They may view uncertainty itself as an unavoidable threat and interpret it more negatively, leading to an inability to tolerate uncertainty. Furthermore, the results from children, adults, and females with substance use disorder showed a positive association between BIS and IU (35–37). Therefore, BIS appears to be an important predictive factor for IU. In contrast, BAS is a motivational system that promotes positive and exploratory behaviors (36). Individuals with higher levels of BAS show a greater tendency to respond to rewards and an increased motivation to engage in reward-seeking behaviors (38). Meanwhile, BAS is associated with positive emotions, and individuals with high levels of BAS may approach life with a more positive attitude (29). When faced with uncertain situations, they are more likely to adopt positive coping styles to reduce the penalties it may bring, which may increase the individual's tolerance for uncertainty (39). In addition, the results from adults provided support for the negative association between BAS and IU (36). Thus, BAS may be a protective factor for IU. On the other hand, IU may have a predictive role in the developmental mechanism of Internet addiction. First, individuals with higher IU are often likely to overestimate threat and underestimate their ability to handle them when coping with uncertain events (40); thus, these individuals experience increased levels of adverse emotions and adopt negative coping styles, such as using the Internet as an avoidance strategy to cope with uncertainty-induced negative experiences (41). Second, a high fear of uncertainty may lead them to seek comfort by spending a great deal of time excessively searching for information and answers on the Internet (42). Previous studies have focused more on the role of IU in mental diseases such as anxiety, depression and obsessive-compulsive disorder (43–45). In recent years, IU has been gradually extended to the field of addiction (such as drug addiction and Internet addiction) (46, 47). However, there are no relevant empirical studies that delve into whether IU plays a mediating role in the relationship between BIS/BAS and Internet addiction.

Self-control is the process by which an individual consciously overcomes impulses, habits or automatic reactions and adjusts his or her behavior to pursue long-term goals (48, 49). First, the level of self-control may be affected by BIS/BAS. When the BIS system is activated, along with increased arousal and attention, there is a disposition for the individual to experience negative emotions (e.g., anxiety and sadness) and to show behavioral inhibition toward desired goals (50). Negative emotions caused by BIS can contribute to a decrease in personal self-control by consuming more cognitive resources (51). In contrast, when the BAS system is activated, individuals have a tendency to experience positive emotions (e.g., hope, happiness) and manifest behavior that helps them approach desired goals (52). In other words, individuals with high levels of BAS may be more likely to respond actively to positive information in their lives and are more willing to control behaviors for which they can receive emotional or material rewards and thus may show higher levels of self-control. In turn, self-control is an important predictor of Internet addiction. High self-control has been found to be closely related to various positive outcomes, such as more stable emotional states and better academic performance (49, 53, 54). However, low self-control was associated with a variety of negative psychological and behavioral problems, such as anxiety, depression, aggressive behavior and addictive behavior (55–57). Cross-sectional and longitudinal studies have shown that self-control plays an important role in the development of Internet addiction (58, 59). People with low self-control lack consideration of the consequences of their actions and tend to act impulsively, which raises the probability of Internet addiction (60, 61). Therefore, self-control may be a mediating variable in the connection between BIS/BAS and Internet addiction. However, to our knowledge, few studies have examined these associations in college student populations.

### The moderating role of gender

Gender is an important demographic factor influencing Internet addiction. Previous studies have shown that there are gender differences in the prevalence of Internet addiction, with males having a relatively higher tendency toward Internet addiction than females (62). However, we should focus not only on the gender differences in the prevalence of Internet addiction, but also on the gender differences in the formation mechanism of Internet addiction. First, it was reported that there may be gender differences in the predictors of Internet addiction. For example, BAS can significantly predict Internet addiction in males, while BIS can significantly predict Internet addiction in females (63). A magnetic resonance imaging (MRI) study showed that females displayed a negative correlation between BIS sensitivity and regional gray matter volume (rGMV) in the parahippocampal gyrus (PHG), as well as positive correlations between BAS sensitivity and rGMV in the ventromedial prefrontal cortex (vmPFC) and inferior parietal lobule (IPL), whereas males showed the opposite pattern, indicating that the relationships between neuroanatomical characteristics and BIS/BAS exhibit sex differences. Meanwhile, individual's BIS/BAS and the effect of BIS/BAS on an individual's cognition, emotion and behavior may be influenced by the individual's genetic, social and other characteristics (64). Then, social role theory provides a theoretical basis for understanding gender differences. It argues that different social divisions of labor can make a difference in the shaping of gender roles (65). Specifically, men are socialized to be strong, rational, and accustomed to solving problems alone, while women are socialized as warm, compassionate, sensitive, and emotionally expressive (66). Thus, when faced with uncertain situations, they may tend to adopt different coping strategies and exhibit different levels of tolerance, which in turn may affect their levels of Internet addiction. In addition, females are asked to manage their behavior effectively in the social contexts, so they may pay a greater emphasis on self-control in their daily activities than males (67). For example, females may be more inclined to avoid losses than males and may have greater self-control (68, 69). The difference in female's and male's ability to control their own behavior may also lead to differences in the formation mechanism of Internet addiction. The above findings provide potential evidence that the mechanism by which Internet addiction develops may be moderated by gender. However, previous studies have focused more on gender differences in a single variable, and the mechanisms underlying these differences have rarely been explored in more realistic and complex models.

### The present study

**Figure 1:**
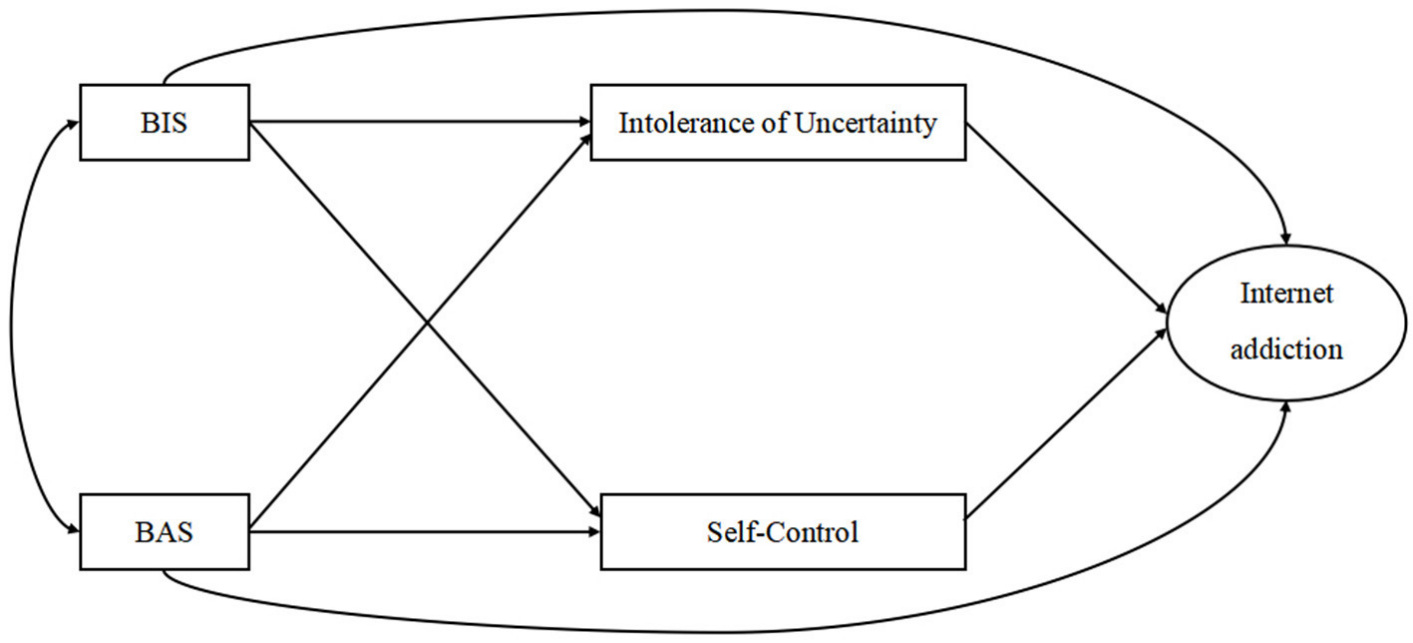
The supposed model.

The aim of this study was to investigate how BIS/BAS influences Internet addiction in college students. Specifically, this study examined the mediating effects of IU and self-control on BIS/BAS and Internet addiction as well as the gender differences among these associations. To the best of our knowledge, this is the first comprehensive empirical study incorporating BIS/BAS, IU, self-control and gender factors and their roles in Internet addiction. On the basis of I-PACE theory, the proposed model is shown in Figure 1. It is reasonable to hypothesize that BIS and BAS act as triggers for individual Internet addiction, IU and self-control serve as mediators between predisposing factors and Internet addiction, and gender plays a moderating role in these associations among college students. More specifically, we propose the following hypotheses: (1) BIS would be positively correlated with Internet addiction, while BAS would be negatively correlated with Internet addiction; (2) IU and self-control would play mediating roles between BIS/BAS and Internet addiction; and (3) the formation mechanism of Internet addiction may be moderated by gender.

## Materials and methods

### Participants

This was a cross-sectional study conducted through an online survey during the COVID-19 pandemic from October 12 to November 8, 2021, when the campus implemented closed-off management to adopt offline learning. All participants were recruited through a convenient cluster sampling method from municipal key universities in Tianjin City, China. In order to ensure the quality of the questionnaire, a questionnaire item (“To check the quality of your response to the questionnaire, please select 2 for this question, which has five answer choices from 1 to 5.”) was included at the end of the survey to reduce the risk of irresponsible answers. In total, 803 students participated in this survey and 56 participants were excluded (45 answered the questionnaire incompletely and 11 failed to answer the questionnaire item for evaluating the quality of survey correctly). Thus, data from 747 participants (*M*_*age*_ = 18.062 years, *SD* = 0.651 years, age range: 16–21 years) were analyzed in this study, including 228 male students (30.5%) and 519 female students (69.5%). Informed consent was obtained from all participants prior to the survey. The study and data collection procedure received approval from the Ethics Committee of Tianjin Medical University (study number: 190002).

### Measures

#### Behavioral inhibition system/behavioral activation system (BIS/BAS) scales

The revised Chinese BIS/BAS Scale is an 18-item questionnaire measured on a 4-point Likert scale from 1 (totally disagree) to 4 (totally agree) (70). The questionnaire includes a 5-item BIS scale (e.g., “If I think something unpleasant is going to happen, I usually get pretty worked up”) and 13-item BAS scale (e.g., “If I see a chance to get something I want, I move on it right away”) (71). The latter scale can be grouped into three subscales: drive (BAS-drive, 4 items), reward responsiveness (BAS-reward, 5 items), and fun seeking (BAS-fun, 4 items). In this study, we used the total score of all 5 BIS items and all 13 BAS items to generate a single BIS score and BAS score, respectively. The scale has been reported to have good reliability and validity among the Chinese population (72). The Cronbach's alpha coefficient was 0.756 for BIS and 0.867 for BAS in the present sample.

#### Intolerance of uncertainty scale (IUS-12)

Intolerance of uncertainty was assessed using the revised Chinese 12-item short-form Intolerance of Uncertainty Scale (73). This scale demonstrates a two-factor structure, evaluating prospective IU (7-item subscale; e.g., “Unforeseen events upset me greatly”), and inhibitory IU (5-item subscale; e.g., “When I am uncertain I can't function very well”) (74). Items are scored on a 5-point Likert scale ranging from 1 (not at all characteristic of me) to 5 (entirely characteristic of me). The total score ranges between 12 and 60. Higher scores indicate higher intolerance of uncertainty. According to reports, the scale has good reliability and validity among Chinese college students (75). In this study, the Cronbach's alpha coefficient was 0.852.

#### Brief self-control scale (BSCS)

The Chinese version of the Brief Self-Control Scale was adopted in this study (76). The BSCS is a questionnaire that assesses one's degree of self-control on a 5-point Likert scale used ranging from 1 (Not at all like me) to 5 (Very much like me). It is composed of 7 items and is divided into two subscales: 3 items measure self-discipline (e.g., “People would say that I have iron self-discipline”) and 4 items measure impulse control (e.g., “I do certain things that are bad for me if they are fun”) (77). The overall self-control score is determined by summing all seven items, with higher scores denoting greater self-control. The Chinese version of the BSCS shows good internal consistency and validity in Chinese individuals (76). In the present study, the Cronbach's alpha coefficient of the BSCS was 0.728.

#### Young's diagnostic questionnaire for Internet addiction

Internet addiction was measured using Young's 8-item diagnostic questionnaire for Internet addiction (5). Responses to each question were provided on a 6-point Likert scale (ranging from “1 = strongly disagree” to “6 = strongly agree”). A sample item is “Have you repeatedly made unsuccessful efforts to control, cut back, or stop Internet use?”. Higher scores reflect a higher degree of Internet addiction. The scale has been used by many researchers and also has good reliability and validity in Chinese adolescents and college students (78, 79). In this study, Cronbach's alpha coefficient for Internet addiction was 0.875.

### Data analysis

First, the common method bias test, descriptive statistics, Pearson's correlation analysis and independent samples *t*-test were examined with SPSS 26.0. Second, Amos 26.0 was used to examine the hypothesized model. Third, a bootstrap procedure was conducted to test the mediating roles of IU and self-control in the relationship between BIS/BAS and Internet addiction. Specifically, the bias-corrected percentile bootstrap method (5,000 bootstrap samples) with 95% confidence intervals (CIs) was performed to examine the significance of mediation effects. CIs excluding zero indicated significant effects. Fourth, participants were split into two groups based on their gender, and a multi-group analysis was used to examine the gender differences in the associations between the variables. In this step, we constructed three nested models, including an unrestricted free estimation model (M0), a model with equal restricted factor loadings (M1), and a model with equal restricted path coefficients (M2). The equivalence of the measurement model across gender was examined by the comparison of M0 and M1. Furthermore, we tested whether there are gender differences in the patterns of association between the variables by comparing M1 and M2.

The chi-square statistic was employed to evaluate the model fit. However, the chi-square statistic is sensitive to sample size (80). Therefore, we used the chi-square to degrees of freedom ratio (χ^2^/*df*) to test model fit. A χ^2^/*df* ratio value below 5 is regarded as an acceptable model fit (81). Meanwhile, the comparative fit index (CFI) (82), Tucker-Lewis index (TLI) (83), standardized root mean square residual (SRMR) (84), and root mean square error of approximation (RMSEA) (85) were also used to evaluate the goodness of fit. The CFI and TLI equal to or above 0.95 and the RMSEA and SRMR lower than 0.08 indicate good model fit (84). For the comparison of the nested models, differences in the χ^2^ (Δχ^2^) and the degrees of freedom (Δ*df*) were used to compare the models with the goodness of fit to determine the model that best fit the data (86, 87). Specifically, the criteria for comparing the two nested models are as follows: When the degrees of freedom increase, but the corresponding chi-square value does not increase significantly (that is, Δχ^2^/Δ*df* is not significant), the model with the higher degrees of freedom is better. Otherwise, the better model is the one with lower degrees of freedom. The predictive and explanatory powers of the model were assessed using path coefficients and *R*^2^.

## Results

### Common method bias test

Participants replied to self-report questionnaires in the present study, which might lead to common method bias. To control this issue, we used the anonymous survey and reverse scores in some questions. Then, common method bias was assessed by Harman's single factor test before processing data (88). The results showed that 11 factors' eigenvalues were larger than 1. The load of the first factor was 17.016%, which was <40% of the covariance among all the items. It suggested that there was no serious common method bias in this study.

### Descriptive statistics and *t* tests

Students' ranges, means and standard deviations of the continuous variables are presented in Table 1. There was a significant difference in age between females and males. The age of females ranged from 16 to 20 years old (*M* = 18.023, *SD* = 0.651) and males ranged from 16 to 21 years old (*M* = 18.153, *SD* = 0.642). These results indicate that males are slightly older than females. In addition, BIS yielded significant gender differences. Compared with males, females had higher scores on the BIS scales. Furthermore, the scores of IU were significantly higher in males than in females. However, the gender differences in Internet addiction, BAS and self-control were not significant.

**Table 1:** Descriptive statistics among the variables.

**Variables**	**Whole sample (*n* = 747)**			**Females (*n* = 519)**			**Males (*n* = 228)**			***t-*test**
	**Range**	***M***	***SD***	**Range**	***M***	***SD***	**Range**	***M***	***SD***	
Age	16–21	18.062	0.651	16–20	18.023	0.651	16–21	18.153	0.642	−2.530^*^
Internet addiction	8–48	20.378	7.888	8–47	20.745	7.631	8–48	19.544	8.402	1.921
BIS	5–20	15.304	2.405	5–20	15.426	2.358	5–20	15.026	2.492	2.095^*^
BAS	13–52	41.815	5.122	13–52	41.703	4.919	19–52	42.070	5.562	−0.901
IU	12–60	27.415	7.613	12–60	27.049	7.308	12–60	28.272	8.218	−2.043^*^
Self-Control	7–35	22.240	4.270	7–35	22.380	4.061	7–35	21.921	4.704	1.352

BIS, Behavioral Inhibition System; BAS, Behavioral Activation System; IU, Intolerance of Uncertainty.

* *p* < 0.05.

### Correlation analyses

Table 2 presents the correlations between variables included in the study. For both males and females, Internet addiction was found to be negatively correlated with both BAS and self-control but IU was shown to have a positive correlation with Internet addiction. Internet addiction was only positively related to BIS in females but had no correlations in males. For both females and males, BIS was significantly positively related to IU while BIS was only negatively related to self-control in females but had no correlations in males. BAS was positively related to self-control but was not significantly related to IU among all participants. In addition, age had no significant correlation with any of the variables included in the study. Furthermore, there were significant gender differences in the correlations based on a one-tailed *z*-test, with a stronger association between Internet addiction and BIS (*z* difference = 2.233, *p* < 0.05) for females than for males, a stronger association between Internet addiction and BAS (*z* difference = 3.191, *p* < 0.001) for males than for females, a stronger association between Internet addiction and IU (*z* difference = −1.779, *p* < 0.05) for males than for females, a stronger association between Internet addiction and self-control (*z* difference = −2.244, *p* < 0.05) for females than for males, and a stronger association between BIS and IU (*z* difference = 2.729, *p* < 0.01) for females than for males. According to the relevant models of these variables, the hypothesized models were analyzed as follows.

**Table 2:** Associations among the variables for females and males.

**Variables**	**1**	**2**	**3**	**4**	**5**	**6**
1. Age	-	−0.033	−0.047	−0.032	−0.038	0.048
2. Internet addiction	−0.083	-	0.215^**^	−0.127^**^	0.224^**^	−0.649^**^
3. BIS	0.124	0.040	-	0.337^**^	0.408^**^	−0.213^**^
4. BAS	0.082	−0.365^**^	0.433^**^	-	0.048	0.219^**^
5. IU	−0.046	0.354^**^	0.212^**^	−0.006	-	−0.168^**^
6. Self-Control	0.103	−0.533^**^	−0.089	0.331^**^	−0.257^**^	-

Females' correlations appear above the diagonal and males' correlations below the diagonal.

** *p* < 0.01.

### Structural equation modeling analyses

**Figure 2:**
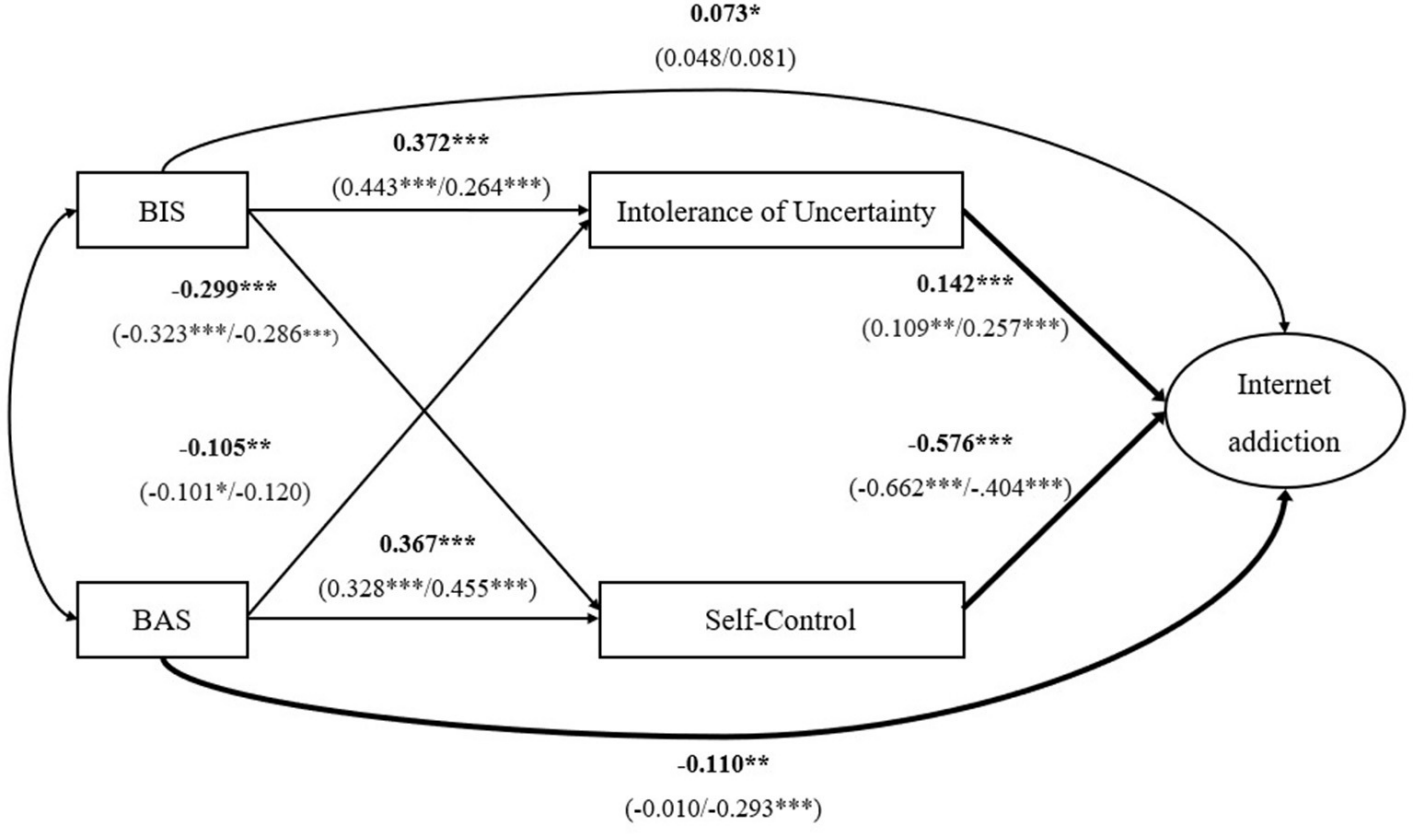
The relationships among BIS, BAS and Internet addiction are mediated by IU and Self-Control. Bold lines indicate significant gender differences in these paths. The parameters for whole participants are displayed outside of the parentheses, while the parameters for different subgroups are denoted within the parentheses (females in the left, males in the right) *p < 0.05, **p < 0.01, ***p < 0.001.

First, the hypothesized model contained four observed variables (BIS, BAS, IU, and self-control, representative of their total scores) and one latent variable (Internet addiction) to make it simpler and more efficient. Moreover, the Young's diagnostic questionnaire for Internet addiction was separated into two parcels, where the sum of odd items constituted the first parcel (parcel 1) and the sum of even items constituted the second parcel (parcel 2), to act as indicators of Internet addiction employing an item-to-construct balance approach (89). Then, structural equation modeling was used to analyze the hypotheses of this study. The standardized factor loadings of Internet addiction for parcel 1 and parcel 2 ranged from 0.866 to 0.913. According to the fit standards, the results of structural equation modeling yielded a good fit to the empirical data, χ^2^ = 16.169, χ^2^/*df* = 4.042, TLI = 0.968, CFI = 0.992, SRMR = 0.032, RMSEA = 0.064. In sum, BIS was positively related to Internet addiction and IU and negatively related to self-control. BAS was negatively related to Internet addiction and IU and positively related to self-control. IU was positively associated with Internet addiction, while self-control was negatively associated with Internet addiction (see Figure 2). Finally, the results of bootstrap analyses showed that the total, direct, and indirect effects of BIS/BAS on Internet addiction were significant (see Table 3). These results indicated that IU and self-control partially mediated the relationships between BIS/BAS and Internet addiction among Chinese college students.

**Table 3:** Total, direct, and indirect effects of BIS/BAS on Internet addiction.

**Effects of predictors**	**β**	**Bias-correlated 95% CI**
**Whole sample**		
BIS		
TE	0.298^***^	[0.222, 0.375]
DE	0.073^*^	[0.004, 0.145]
IE-IU	0.053^***^	[0.027, 0.083]
IE-SC	0.172^***^	[0.126, 0.222]
BAS		
TE	−0.336^***^	[−0.425, −0.251]
DE	−0.110^**^	[−0.185, −0.039]
IE-IU	−0.015^**^	[−0.032, −0.005]
IE-SC	−0.211^***^	[−0.268, −0.160]
**Females**		
BIS		
TE	0.311^***^	[0.218, 0.397]
DE	0.048	[−0.036, 0.128]
IE-IU	0.048^**^	[0.013, 0.091]
IE-SC	0.214^***^	[0.150, 0.281]
BAS		
TE	−0.238^***^	[−0.340, −0.141]
DE	−0.010	[−0.087, 0.065]
IE-IU	−0.011^*^	[−0.029, −0.002]
IE-SC	−0.217^***^	[−0.287, −0.149]
**Males**		
BIS		
TE	0.264^**^	[0.106, 0.417]
DE	0.081	[−0.044, 0.218]
IE-IU	0.068^**^	[0.031, 0.127]
IE-SC	0.116^***^	[0.054, 0.191]
BAS		
TE	−0.508^***^	[−0.656, −0.355]
DE	−0.293^***^	[−0.429, −0.157]
IE-IU	−0.031	[−0.078, 0.004]
IE-SC	−0.184^***^	[−0.276, −0.111]

All the estimates provided in the table are standardized estimates. TE, total effect; DE, direct effect; IE, indirect effect; IU, intolerance of uncertainty; SC, self-control; CI, confidence interval.

* *p* < 0.05,

** *p* < 0.01,

*** *p* < 0.001.

Cross-group comparison was next used to examine the gender differences present in the model. First, the result of the comparison of M0 and M1, Δχ^2^ (1) = 0.288, *p* > 0.05, indicated that there was measurement equivalence between female and male groups. Second, to examine whether the relationship of each path differed across gender, two nested models (M1 and M2) were tested. The results demonstrated that these two models were significantly different, Δχ^2^ (8) = 32.432, *p* < 0.001, indicating that their path coefficients differed across gender. Next, the critical ratios of differences (CRDs) were calculated to test for differences between both groups among structural path coefficients. If the absolute value of CRD was equal to or greater than 1.96, then the association between these two variables were considered to be a significant gender difference at the *p* < 0.05 level. The results indicated that the relationships between BAS and Internet addiction and between IU and Internet addiction were stronger among male participants. The relationship between self-control and Internet addiction was stronger among female participants (see Table 4). Finally, we found that 49.0% of the variance in Internet addiction could be explained among females and 40.8% of the variance in Internet addiction could be explained among males by this model.

**Table 4:** Standardized coefficients from the multiple–group analysis.

**Structural model**	**Female estimate (*S.E*.)**	**Male estimate (*S.E*.)**	**CRD**
BIS to Internet addiction	0.048 (*0.017*)	0.081 (*0.027*)	−0.433
BAS to Internet addiction	−0.010 (*0.008*)	−0.293 (*0.013*)^***^	3.539^*^
BIS to intolerance of uncertainty	0.443 (*0.131*)^***^	0.264 (*0.236*)^***^	1.860
BIS to Self-Control	−0.323 (*0.075*)^***^	−0.286 (*0.126*)^***^	−0.112
BAS to intolerance of uncertainty	−0.101 (*0.063*)^*^	−0.120 (*0.106*)	0.216
BAS to Self-Control	0.328 (*0.036*)^***^	0.455 (*0.056*)^***^	−1.713
Intolerance of uncertainty to Internet addiction	0.109 (*0.005*)^**^	0.257 (*0.007*)^***^	−2.028^*^
Self-Control to Internet addiction	−0.662 (*0.009*)^***^	−0.404 (*0.014*)^***^	−4.008^*^

The numbers in italics in the parentheses represent the standard errors. S.E., standard error; CRD, critical ratio difference. *p < 0.05, **p < 0.01, ***p < 0.001.

## Discussion

To our knowledge, this is the first study to test the impact mechanism of BIS/BAS on Internet addiction through IU and self-control among Chinese college students by applying the I-PACE model and further consider the moderating role of gender among these variables. These results indicated that BIS was positively correlated with the level of Internet addiction, while BAS was negatively related to the level of Internet addiction of college students. Moreover, IU and self-control had statistically significant mediating effects between BIS/BAS and Internet addiction. Specifically, BAS and IU showed significantly greater predictive abilities for Internet addiction in males than in females, while the protective role of self-control on Internet addiction was greater in females than in males. Overall the results supported the I-PACE model that specific personal characteristics (e.g., BIS/BAS), affective and cognitive responses (e.g., IU), executive function (e.g., self-control) components resulted in adverse emotional reactions through the perception of the situation and led to certain addictive tendencies (17). The findings of this study extend previous studies about the effect of BIS/BAS on Internet addiction, and explore the mediation mechanisms and gender differences among these variables, which are helpful for understanding the mechanism of Internet addiction more accurately and providing more effective guidance for the Internet addiction intervention among college students.

### Associations between BIS/BAS and Internet addiction

In line with our speculation, Internet addiction has a significant positive correlation with BIS and a significant negative relationship with BAS. First, prior research showed that individuals who have higher BIS levels are more sensitive to punishment stimuli and have a higher risk of Internet addiction (63). Individuals with high BIS may have more avoidance behaviors in real life due to fear of failure, and they may have anxiety and distress in face-to-face interpersonal interaction, preventing them from communicating normally. However, they can communicate anonymously to meet their psychological needs that cannot be met in real life and feel a sense of achievement and comfort in a virtual world. This experience would significantly increase the Internet use time, thereby increasing the possibility of Internet addiction (90). Next, college students who have higher BAS levels are more sensitive to reward stimuli and have a lower risk of Internet addiction (23). College is a vital stage of life development and learning plays a leading role in college life. During this period, students will strive to learn knowledge and improve their abilities to pursue a better career. Previous research suggests that BAS may play an important role in individuals' motivations to conduct goal-directed behavior (91, 92). Therefore, students with high BAS may aim for behaviors that benefit their developmental plans, and spend more time on positive choices that benefit growth and less time on negative behaviors that hinder growth, which may reduce the likelihood of Internet addiction. In addition, individuals with high BAS sensitivity tend to approach novel people or things, and they may invest more in social activities and obtain higher happiness (93), which further makes individuals less prone to Internet addiction behaviors.

### IU and self-control as mediators

On the one hand, there was a mediating role of IU between BIS/BAS and Internet addiction. First, BIS can reinforce individual reactions to avoid or withdraw from novel, uncertain or threatening situations, which may trigger individuals to interpret unknown events more negatively (34). This is especially true for students at the college stage, who face constant academic and competency assessment and assume greater uncertainty about their future due to the rapid development of society and increased competition. Therefore, individuals with high BIS levels may experience higher levels of IU than individuals with low BIS levels. Second, BAS is closely related to reward seeking, and individuals with high-BAS sensitivity have a higher preference for novelty processing and reward dependence (94). They are highly motivated by both external and internal rewards and are able to devote themselves to an activity in which they feel a sense of pleasure and control (95). Thus, high-BAS individuals may have a higher desire to explore perceived goals, and uncertain situations may be perceived as novel and rewarding stimuli. When faced with an unknown situation, they may be more likely to choose to actively engage in exploring the environment and constantly challenge themselves compared to reacting with aversion and avoidance, which may increase their tolerance for uncertainty to some extent. Third, individuals with high IU tend to have more negative thinking patterns, believing that they lack sufficient coping or problem-solving skills to effectively face future negative outcomes, which may cause discomfort and negative emotions in individuals (96). To eliminate adverse experiences, individuals may seek temporary happiness on the Internet or increase the certainty of knowledge of unknowable events through excessive searching for information. However, these actions can only temporarily reduce anxiety and distress which may lead to heightened perceptions of uncertainty and anxiety in the long run (97). Especially, the repeated COVID-19 epidemic has increased the uncertainty of the current living environment and future development of college students (98). The perception of these serious uncertainties may reduce their tolerance for uncertainty, which also causes them to spend more time on the Internet and inadvertently further exacerbates the risk of Internet addiction among college students.

On the other hand, the results demonstrated that self-control serves as a mediator between BIS/BAS and Internet addiction and contributes to explaining how BIS/BAS influences Internet addiction. First, BIS was significantly negatively related to self-control, while BAS was significantly positively correlated with self-control. According to the strength model of self-control, an individual's emotional state has an important impact on the ability to control oneself. Negative emotions weaken self-control by depleting more cognitive resources, while positive emotions facilitate the recovery and maintenance of self-control resources (99, 100). Hence, negative emotions such as anxiety generated by high BIS activation may decrease the level of self-control, whereas positive emotions experienced during high BAS activation may allow individuals to maintain a higher level of self-control. Second, self-control was negatively linked to Internet addiction among college students. In general, individuals with low self-control are more likely to experience problem behaviors than those with high self-control (101–103). This may be because people with high self-control have a greater ability to delay gratification, reduce the impact of temptations in the moment, and restrain themselves to achieve greater satisfaction (104). However, individuals with low self-control tend to be highly impulsive and often choose to experience instant pleasure online when faced with the lure of the Internet (105). Especially during the COVID-19 epidemic, entertainment options became limited, and the online world became one of the few entertainment options available to college students, which may increase the risk of Internet addiction in college students with low self-control.

### Gender differences in the associations between BIS/BAS, IU, self-control and Internet addiction

The present study further illuminated gender differences in the strength of the associations between the variables being studied, and the findings support the third hypothesis that the formation mechanism of Internet addiction are moderated by gender. First, the direct predictive effect of BAS on Internet addiction was significant for males but non-significant for females. Meanwhile, the association between IU and Internet addiction was stronger in males than in females. In other words, Internet addiction in males was more likely to be influenced by BAS and IU. The social role theory indicates that men being expected to be independent and brave, to focus on the outside world, and to achieve academic and career success (65). As a result, males may be more motivated to choose positive behaviors and set higher goals for self-growth than females, which enables males with high BAS to use the Internet appropriately and be less prone to Internet addiction. In addition, most parents have a higher aptitude and educational expectations of men (106). Male college students who have high expectations place more value on these achievements and are more concerned about the adverse effects of poor performance on their future career prospects. Earlier research has identified that when dealing with negative emotions, females can seek social support and use effective emotion regulation strategies while males are more prone to suppress or avoid emotional expression (107). This puts male college students with high IU at a higher risk of Internet addiction as they are likely to spend substantial amounts of time online. Second, the association between self-control and Internet addiction is more powerful among female college students than among male students. Females are more likely to have inappropriate behaviors monitored, identified, and corrected by their parents than males during the learning process of socialization (108), which may make self-control play a more important role in females than in males. Thus, females with high self-control may have strict demands on their study and life, and can show strong control in the face of temptation-filled behaviors and undesirable behaviors such as Internet addiction. Previous studies have shown that males and females differ in their preferences for Internet use (109). Males prefer to play games and watch videos online, while females prefer communication features and social networking services online. However, when females have low self-control, they may also choose online games for entertainment and relaxation because the virtual world of the Internet is always a temptation for people (110, 111). In addition, it was reported that females prefer online shopping features over males and that the diversity of online products may now be more skewed toward females (112). Thus, females with low self-control may also be more likely to spend too much time on the Internet than males with low self-control (113).

### Implications for theory and practice

From a theoretical perspective, this study supplements and expands previous studies on the influence mechanism of BIS/BAS on Internet addiction among college students. In addition, it provides empirical support for the I-PACE model of Internet addiction among college students in the context of Chinese culture as well as a reference for further research on the formation mechanism of Internet addiction. From a practical point of view, our findings may be helpful for guiding the prevention and intervention of Internet addiction among college students. First of all, when screening and choosing a target population, the population with high BIS, low BAS, high IU and low self-control should be of particular concern. Second, to prevent and intervene in Internet addiction in college students, training techniques should be used to improve students' tolerance of uncertainty and self-control ability because improving specific behaviors may be more efficient than directly changing personality (114). On the one hand, cognitive behavioral therapy techniques for IU (CBT-IU) can help college students with high BIS and low BAS to improve their tolerance for uncertainty through worry awareness training, cognitive reappraisal of uncertainty, and other methods (43). On the other hand, mindfulness-based stress reduction techniques and general self-control intervention in a cognitive behavioral framework can have a positive impact on the self-control ability of college students with Internet addiction (115, 116). Finally, more attention should be given to developing gender-specific Internet addiction prevention and intervention programs for male and female college students. For females, low self-control is the most critical factor contributing to their Internet addiction. Therefore, the prevention and intervention of Internet addiction for them should focus on the improvement of self-control. For males, apart from low self-control, low BAS and high IU also play important roles in the formation mechanism of Internet addiction. Thus, in addition to self-control enhancement training, more targeted guidance or training should be given to those with low BAS levels and high IU levels to reduce the occurrence and development of Internet addiction.

### Limitations and further directions

Several limitations of the current study should be noted. First, this was a cross-sectional study. Although there is a certain theoretical basis, the research results can only provide the predictions of the relationships among these variables. The cross-sectional design of the study naturally limits any causal interpretation. It is also possible that people with different levels of IU and self-control may show various levels of BIS or BAS, which may further relate to Internet addiction. Thus, future studies should attempt to use longitudinal design and clinical trials to examine the causal relationship and mediating effect between BIS/BAS and Internet addiction. Second, all data were collected through self-report questionnaires, so recall bias and the subject-expectancy effect may be difficult to avoid. Future measures of peer nomination and behavioral tasks could be added, and data could be collected from multiple sources of information to make the data more realistic and reliable. Third, this study did not distinguish Internet addiction into specific subtypes. Previous researches have shown that it is important to distinguish generalized Internet addiction from specific Internet addiction and that there are significant gender differences between online game addiction and social media addiction (117, 118). Therefore, more information on Internet use needs to be included in future studies to verify whether the current findings are appropriate for generalized Internet addiction or specific Internet addiction. Fourth, this study only explores the formation mechanism of Internet addiction from the I-PACE model, but other theoretical approaches exist to view addiction from a different perspective. For example, the model of addiction based on affective neuroscience describes and interprets the phenomenon of addiction as an expression of vulnerability related to biological and social factors from a neuro-psycho-evolutionary perspective (119, 120). Future research should consider combining these different theories in a rigorous manner to further illuminate the underlying mechanism of Internet addiction. Finally, participants were all college students from China. Considering the role of cultural factors on individual psychology and behavior, the model should be tested using different cultures, including collectivist and individualist societies.

## Conclusion

In summary, this study explored the relationship between BIS/BAS and Internet addiction among Chinese college students by using the I-PACE model. The current study indicated that the BIS/BAS shows important association with Internet addiction, that BIS/BAS has indirect influence on Internet addiction through IU and self-control and that the relationships among these variables can also be moderated by gender. Specifically, Internet addiction is more likely to be negatively affected by low BAS and high IU in male college students, while high self-control has a greater potential protective effect on Internet addiction in female students. Given that Internet addiction is not only harmful to the physical and mental health of college students, but also places a large burden on their families and society, this study suggests that policy-makers should further implement strict regulatory measures (e.g., setting up anti-addiction or fatigue system) and actively guide college students to use the Internet and social networks rationally (121). Moreover, educators should combine IU intervention measures and self-control training when formulating prevention and intervention programs for Internet addiction among college students and adopt differentiated programs for males and females in the specific implementation process.

## Data availability statement

The raw data supporting the conclusions of this article will be made available by the authors, without undue reservation.

## Ethics statement

The studies involving human participants were reviewed and approved by the Ethics Committee of Tianjin Medical University. The patients/participants provided their written informed consent to participate in this study.

## Author contributions

BD, ZZ, and ZP designed the study and wrote the protocol. YL, XH, and BD collected the research data. YL, ZZ, BD, and ZP conducted the statistical analyses and wrote the manuscript. JL, GZ, and XH conducted the literature searches, created the figures, and proofread the language. All authors approved the final version of the manuscript.

## References

[1] Kemp S. Digital 2022: Global Overview Report. (2022). Available online at: https://datareportal.com/reports/digital-2022-global-overview-report (accessed August 15, 2022).

[2] Hussain Z Pontes HM. Personality, internet addiction, and other technological addictions: an update of the research literature. In: Bozoglan B editor. Multifaceted Approach to Digital Addiction and Its Treatment. Hershey, PA: IGI Global (2019). p. 46–72.

[3] China Internet Network Information Center [CNNIC]. The 50th Statistical Report on Internet Development in China. (2022). Available online at: http://www.cnnic.net.cn/gywm/xwzx/rdxw/20172017_7086/202208/t20220831_71823.htm (accessed September 1, 2022).

[4] Liang L Zhou D Yuan C Shao A Bian Y. Gender differences in the relationship between internet addiction and depression: a cross-lagged study in Chinese adolescents. Comput Human Behav. (2016) 63:463–70. 10.1016/j.chb.2016.04.04335114630

[5] Young KS. Internet addiction: The emergence of a new clinical disorder. Cyberpsychol Behav. (1998) 1:237–44. 10.1089/cpb.1998.1.237

[6] Sevelko K Bischof G Bischof A Besser B John U Meyer C. The role of self-esteem in Internet addiction within the context of comorbid mental disorders: findings from a general population-based sample. J Behav Addict. (2018) 7:976–84. 10.1556/2006.7.2018.130 30585501 PMC6376382

[7] Alimoradi Z Lin C-Y Broström A Bülow PH Bajalan Z Griffiths MD. Internet addiction and sleep problems: a systematic review and meta-analysis. Sleep Med Rev. (2019) 47:51–61. 10.1016/j.smrv.2019.06.004 31336284

[8] Lin YJ Hsiao RC Liu TL Yen CF. Bidirectional relationships of psychiatric symptoms with internet addiction in college students: a prospective study. J Formos Med Assoc. (2020) 119:1093–100. 10.1016/j.jfma.2019.10.006 31653577

[9] Wang J Hao Q Tu Y Peng W Wang Y Li H. Assessing the association between internet addiction disorder and health risk behaviors among adolescents and young adults: a systematic review and meta-analysis. Front Public Health. (2022) 10:809232. 10.3389/fpubh.2022.809232 35433568 PMC9010676

[10] Cheng Y-S Tseng P-T Lin P-Y Chen T-Y Stubbs B Carvalho AF. Internet addiction and its relationship with suicidal behaviors: a meta-analysis of multinational observational studies. J Clin Psychiatry. (2018) 79:17r11761. 10.4088/JCP.17r11761 29877640

[11] Hsieh K-Y Hsiao RC Yang Y-H Lee K-H Yen C-F. Relationship between self-identity confusion and internet addiction among college students: the mediating effects of psychological inflexibility and experiential avoidance. Int J Environ Res Public Health. (2019) 16:3225. 10.3390/ijerph16173225 31484435 PMC6747481

[12] Gavurova B Ivankova V Rigelsky M Mudarri T. Internet addiction in socio-demographic, academic, and psychological profile of college students during the COVID-19 Pandemic in the Czech Republic and Slovakia. Front Public Health. (2022) 10:944085. 10.3389/fpubh.2022.944085 35812472 PMC9260220

[13] Rogier G Zobel SB Velotti P. COVID-19, loneliness and technological addiction: Longitudinal data. J Gambl Issues. (2021) 47:108–20. 10.4309/jgi.2021.47.4

[14] Lemenager T Neissner M Koopmann A Reinhard I Georgiadou E Müller A. COVID-19 lockdown restrictions and online media consumption in Germany. Int J Environ Res Public Health. (2020) 18:14. 10.3390/ijerph18010014 33375139 PMC7792961

[15] Pan Y-C Chiu Y-C Lin Y-H. Systematic review and meta-analysis of epidemiology of internet addiction. Neurosci Biobehav Rev. (2020) 118:612–22. 10.1016/j.neubiorev.2020.08.013 32853626

[16] Shao Y-j Zheng T Wang Y-q Liu L Chen Y Yao Y-s. Internet addiction detection rate among college students in the People's Republic of China: a meta-analysis. Child Adolesc Psychiatry Ment Health. (2018) 12:1–10. 10.1186/s13034-018-0231-6 29849754 PMC5970523

[17] Brand M Young KS Laier C Wölfling K Potenza MN. Integrating psychological and neurobiological considerations regarding the development and maintenance of specific Internet-use disorders: an interaction of person-affect-cognition-execution (I-PACE) model. Neurosci Biobehav Rev. (2016) 71:252–66. 10.1016/j.neubiorev.2016.08.033 27590829

[18] Brand M Wegmann E Stark R Müller A Wölfling K Robbins TW. The interaction of person-affect-cognition-execution (I-PACE) model for addictive behaviors: Update, generalization to addictive behaviors beyond internet-use disorders, and specification of the process character of addictive behaviors. Neurosci Biobehav Rev. (2019) 104:1–10. 10.1016/j.neubiorev.2019.06.032 31247240

[19] Gray JA. Framework for a taxonomy of psychiatric disorder. In: Goozen SHMV Poll N and Sergeant JA, editors. Emotions: Essays on Emotion Theory. Hillsdale, NJ: Lawrence Erlbaum (1994). p. 29–59.

[20] Hewig J Hagemann D Seifert J Naumann E Bartussek D. The relation of cortical activity and BIS/BAS on the trait level. Biol Psychol. (2006) 71:42–53. 10.1016/j.biopsycho.2005.01.006 16360880

[21] Nam CR Lee DH Lee JY Choi A Chung SJ Kim DJ. The role of resilience in internet addiction among adolescents between sexes: a moderated mediation model. J Clin Med. (2018) 7:222. 10.3390/jcm7080222 30126239 PMC6111304

[22] Zhang L Hou X. Relationships between reinfor cement sensitivity, internet service hobby and pathological internet use in junior middle school students. Chin J Clin Psychol. (2008) 16:164–6. 10.16128/j.cnki1005-3611.2008.02.024

[23] Xiang H Tian X Zhou Y Chen J Potenza MN Zhang Q. The relationship between behavioral inhibition and behavioral activation systems, impulsiveness, and internet gaming disorder among students of different ages. Front Psychiatry. (2021) 11:1546. 10.3389/fpsyt.2020.560142 33510653 PMC7835792

[24] Haller SP Cohen Kadosh K Scerif G Lau JY. Social anxiety disorder in adolescence: How developmental cognitive neuroscience findings may shape understanding and interventions for psychopathology. Dev Cogn Neurosci. (2015) 13:11–20. 10.1016/j.dcn.2015.02.002 25818181 PMC6989773

[25] Dong H Zheng H Wang M Ye S Dong GH. The unbalanced behavioral activation and inhibition system sensitivity in internet gaming disorder: evidence from resting-state Granger causal connectivity analysis. Prog Neuropsychopharmacol Biol Psychiatry. (2022) 119:110582. 10.1016/j.pnpbp.2022.110582 35661790

[26] Yen JY Cheng-Fang Y Chen CS Chang YH Yeh YC Ko CH. The bidirectional interactions between addiction, behaviour approach and behaviour inhibition systems among adolescents in a prospective study. Psychiatry Res. (2012) 200:588–92. 10.1016/j.psychres.2012.03.015 22534501

[27] Franken IH Muris P Georgieva I. Gray's model of personality and addiction. Addict Behav. (2006) 31:399–403. 10.1016/j.addbeh.2005.05.022 15964149

[28] Magnani G Zucchella A. Coping with uncertainty in the internationalisation strategy: an exploratory study on entrepreneurial firms. Int Market Rev. (2018) 36:131–63. 10.1108/IMR-02-2017-0042

[29] Gable SL Reis HT Elliot AJ. Behavioral activation and inhibition in everyday life. J Pers Soc Psychol. (2000) 78:1135–49. 10.1037/0022-3514.78.6.1135 10870914

[30] Rosser BA. Intolerance of uncertainty as a transdiagnostic mechanism of psychological difficulties: a systematic review of evidence pertaining to causality and temporal precedence. Cognit Ther Res. (2019) 43:438–63. 10.1007/s10608-018-9964-z

[31] Sadeh N Bredemeier K. Engaging in risky and impulsive behaviors to alleviate distress mediates associations between intolerance of uncertainty and externalizing psychopathology. J Pers Disord. (2021) 35:393–408. 10.1521/pedi_2019_33_456 31682196 PMC8314479

[32] Dugas MJ Schwartz A Francis K. Brief report: intolerance of uncertainty, worry, and depression. Cognit Ther Res. (2004) 28:835–42. 10.1007/s10608-004-0669-0

[33] Sheynin J Beck KD Pang KCH Servatius RJ Shikari S Ostovich J. Behaviourally inhibited temperament and female sex, two vulnerability factors for anxiety disorders, facilitate conditioned avoidance (also) in humans. Behav Processes. (2014) 103:228–35. 10.1016/j.beproc.2014.01.003 24412263 PMC3972301

[34] Radell ML Myers CE Beck KD Moustafa AA Allen MT. The personality trait of intolerance to uncertainty affects behavior in a novel computer-based conditioned place preference task. Front Psychol. (2016) 7:1175. 10.3389/fpsyg.2016.01175 27555829 PMC4977360

[35] Zdebik MA Moss E Bureau JF. Childhood attachment and behavioral inhibition: predicting intolerance of uncertainty in adulthood. Dev Psychopathol. (2018) 30:1225–38. 10.1017/S0954579417001614 29157325

[36] Mihić L Colović P Ignjatović I Smederevac S Novović Z. Anxiety between personality and cognition: the gray zone. Pers Individ Dif. (2015) 78:19–23. 10.1016/j.paid.2015.01.013

[37] Xie J Fang P Zhang Z Luo R Dai B. Behavioral inhibition/activation systems and depression among females with substance use disorder: the mediating role of intolerance of uncertainty and anhedonia. Front Psychiatry. (2021) 12:644882. 10.3389/fpsyt.2021.644882 33746802 PMC7969652

[38] Wadhwa M Shiv B Nowlis SM. A bite to whet the reward appetite: the influence of sampling on reward-seeking behaviors. J Mark Res. (2008) 45:403–13. 10.1509/jmkr.45.4.403

[39] Hong RY Lee SSM. Further clarifying prospective and inhibitory intolerance of uncertainty: factorial and construct validity of test scores from the intolerance of uncertainty scale. Psychol Assess. (2015) 27:605–20. 10.1037/pas0000074 25602690

[40] Reuman L Jacoby RJ Fabricant LE Herring B Abramowitz JS. Uncertainty as an anxiety cue at high and low levels of threat. J Behav Ther Exp Psychiatry. (2015) 47:111–9. 10.1016/j.jbtep.2014.12.002 25562749

[41] Zhang G Dai B. A summary of research on intolerance of uncertainty. J Cap Normal Univ. (2012) 205:124–30.

[42] Du G Lyu H. Future expectations and internet addiction among adolescents: the roles of intolerance of uncertainty and perceived social support. Front Psychiatry. (2021) 12:727106. 10.3389/fpsyt.2021.727106 34512423 PMC8426547

[43] Gillett CB Bilek EL Hanna GL Fitzgerald KD. Intolerance of uncertainty in youth with obsessive-compulsive disorder and generalized anxiety disorder: a transdiagnostic construct with implications for phenomenology and treatment. Clin Psychol Rev. (2018) 60:100–8. 10.1016/j.cpr.2018.01.007 29426573

[44] Del Valle MV Andrés ML Urquijo S Yerro-Avincetto M López-Morales H Canet-Juric L. Intolerance of uncertainty over COVID-19 pandemic and its effect on anxiety and depressive symptoms. Interamerican J Psychol. (2020) 54:e1335. 10.30849/ripijp.v54i2.1335

[45] Freeston MH Rhéaume J Letarte H Dugas MJ Ladouceur R. Why do people worry? Pers Individ Dif. (1994) 17:791–802. 10.1016/0191-8869(94)90048-5

[46] Rozgonjuk D Elhai JD Täht K Vassil K Levine JC Asmundson GJG. Non-social smartphone use mediates the relationship between intolerance of uncertainty and problematic smartphone use: evidence from a repeated-measures study. Comput Human Behav. (2019) 96:56–62. 10.1016/j.chb.2019.02.013

[47] Favaloro B Moustafa AA. Intolerance of uncertainty and addiction. In: Moustafa AA editor. Cognitive, Clinical, and Neural Aspects of Drug Addiction: Academic Press (2020). p. 205–220.

[48] Carver CS Scheier MF. Control processes and self-organization as complementary principles underlying behavior. Pers Soc Psychol Rev. (2002) 6:304–15. 10.1207/S15327957PSPR0604_05

[49] Tangney JP Boone AL Baumeister RF. High Self-Control Predicts Good Adjustment, Less Pathology, Better Grades, and Interpersonal Success. Self-Regulation and Self-Control. London, UK: Routledge (2018). p. 173–212. 10.1111/j.0022-3506.2004.00263.x 15016066

[50] Smillie LD Pickering AD Jackson CJ. The new reinforcement sensitivity theory: implications for personality measurement. Pers Soc Psychol Rev. (2006) 10:320–35. 10.1207/s15327957pspr1004_3 17201591

[51] Heatherton TF Wagner DD. Cognitive neuroscience of self-regulation failure. Trends Cogn Sci. (2011) 15:132–9. 10.1016/j.tics.2010.12.005 21273114 PMC3062191

[52] Taubitz LE Pedersen WS Larson CL. BAS reward responsiveness: a unique predictor of positive psychological functioning. Pers Individ Dif. (2015) 80:107–12. 10.1016/j.paid.2015.02.029 30034067 PMC6053059

[53] Duckworth AL Taxer JL Eskreis-Winkler L Galla BM Gross JJ. Self-control and academic achievement. Annu Rev Psychol. (2019) 70:373–99. 10.1146/annurev-psych-010418-103230 30609915

[54] Troll ES Friese M Loschelder DD. How students' self-control and smartphone-use explain their academic performance. Comput Human Behav. (2021) 117:106624. 10.1016/j.chb.2020.106624

[55] Blase K Vermetten E Lehrer P Gevirtz R. Neurophysiological approach by self-control of your stress-related autonomic nervous system with depression, stress and anxiety patients. Int J Environ Res Public Health. (2021) 18:3329. 10.3390/ijerph18073329 33804817 PMC8036915

[56] Chen X Zhang G Yin X Li Y Cao G Gutiérrez-García C. The relationship between self-efficacy and aggressive behavior in boxers: the mediating role of self-control. Front Psychol. (2019) 10:212. 10.3389/fpsyg.2019.00212 30837910 PMC6389640

[57] Oliva A Antolín-Suárez L Rodríguez-Meirinhos A. Uncovering the link between self-control, age, and psychological maladjustment among spanish adolescents and young adults. Psychosoc Intervent. (2019) 28:49–55. 10.5093/pi2019a1

[58] Agbaria Q. Internet addiction and aggression: the mediating roles of self-control and positive affect. Int J Ment Health Addict. (2021) 19:1227–42. 10.1007/s11469-019-00220-z

[59] Gu M. A longitudinal study of daily hassles, internet expectancy, self-control, and problematic internet use in Chinese adolescents: a moderated mediation model. Pers Individ Dif. (2020) 152:109571. 10.1016/j.paid.2019.109571

[60] Li S Ren P Chiu MM Wang C Lei H. The relationship between self-control and internet addiction among students: a meta-analysis. Front Psychol. (2021) 12:755. 10.3389/fpsyg.2021.735755 34899477 PMC8653951

[61] Agbaria Q Bdier D. The role of self-control and identity status as predictors of internet addiction among Israeli-Palestinian college students in Israel. Int J Ment Health Addict. (2021) 19:252–66. 10.1007/s11469-019-00172-4

[62] Su W Han X Jin C Yan Y Potenza MN. Are males more likely to be addicted to the internet than females? a meta-analysis involving 34 global jurisdictions. Comput Human Behav. (2019). 10.1016/j.chb.2019.04.021

[63] Li Q Dai W Zhong Y Wang L Dai B Liu X. The mediating role of coping styles on impulsivity, behavioral inhibition/approach system, and internet addiction in adolescents from a gender perspective. Front Psychol. (2019) 10:2402. 10.3389/fpsyg.2019.02402 31708840 PMC6821786

[64] Hyde JS Mezulis AH. Gender differences in depression: biological, affective, cognitive, and sociocultural factors. Harv Rev Psychiatry. (2020) 28:4–13. 10.1097/HRP.0000000000000230 31913978

[65] Eagly AH Wood W. Social role theory. In: Lange PAMV Kruglanski AW and Higgins ET editors. Handbook of Theories of Social Psychology. Thousand Oaks, CA: Sage (2012). p. 458–476. Available online at: https://www.torrossa.com/en/resources/an/4912667#page=1065

[66] Reevy GM Maslach C. Use of social support: gender and personality differences. Sex Roles. (2001) 44:437–59. 10.1023/A:101193012882929953517

[67] Luo L Ming H Tian Y Xia X Huang S. The correlations between parenting style and the sense of social responsibility among undergraduate: the mediating role of self-control and the gender difference. Psychol Dev Educ. (2018) 34:164–70.

[68] Lam D Ozorio B. The effect of prior outcomes on gender risk-taking differences. J Risk Res. (2013) 16:791–802. 10.1080/13669877.2012.73782420619505

[69] Wijaya HE Tori AR. Exploring the role of self-control on student procrastination. Int J Res Couns Educ. (2018) 2:13–8. 10.24036/003za000235459983

[70] Li Y-Z Zhang Y Jiang Y Li H Mi S Yi G-J. The Chinese version of the BIS/BAS scale: reliability and validity. Chin Ment Health J. (2008) 22:613–5. 10.3724/SP.J.1041.2008.00418

[71] Carver CS White TL. Behavioral inhibition, behavioral activation, and affective responses to impending reward and punishment: the BIS/BAS scales. J Pers Soc Psychol. (1994) 67:319–33. 10.1037/0022-3514.67.2.319

[72] Yao Q Yue G-a Wu C-c Li Y-f Chen C. Measurement of regulatory focus: the reliability and validity of Chinese version of regulatory focus questionnaire. Chin J Appl Psychol. (2008) 14:318–23.

[73] Zhang Y Song J Gao Y Wu S Song L Miao D. Reliability and validity of the intolerance of uncertainty scale-short form in university students. Chin J Clin Psychol. (2017) 25:285–8. 10.3760/cma.j.issn.1674-6554.2013.10.027

[74] Carleton RN Norton MAPJ Asmundson GJG. Fearing the unknown: a short version of the intolerance of uncertainty scale. J Anxiety Disord. (2007) 21:105–17. 10.1016/j.janxdis.2006.03.014 16647833

[75] Zhang X Zhang S Sun W Wang L Song L Zhao J. The association between intolerance of uncertainty and college students'hoarding behavior: a mediated moderation model. Chin J Clin Psychol. (2020) 28:773–8. 10.16128/j.cnki.1005-3611.2020.04.026

[76] Luo T Cheng LM Qin LX Xiao SY. Reliability and validity of chinese version of brief self-control scale. Chin J Clin Psychol. (2021) 29:83–6. 10.16128/j.cnki.1005-3611.2021.01.017

[77] Morean ME DeMartini KS Leeman RF Pearlson GD Anticevic A Krishnan-Sarin S. Psychometrically improved, abbreviated versions of three classic measures of impulsivity and self-control. Psychol Assess. (2014) 26:1003–20. 10.1037/pas0000003 24885848 PMC4152397

[78] Liu DN Li DP. Parenting styles and adolescent internet addiction: an examination of the mediating and moderating roles of ego-resiliency. J Psychol Sci. (2017) 40:1385–91. 10.16719/j.cnki.1671-6981.20170617

[79] Wei H Duan H Zhou Z Ping F Ding Q. The effect of childhood abuse on internet addiction: moderated mediating effect. Psychol Dev Educ. (2020) 36:77–83. 10.16187/j.cnki.issn1001-4918.2020.01.09

[80] Bollen KA. A new incremental fit index for general structural equation models. Sociol Methods Res. (1989) 17:303–16. 10.1177/0049124189017003004

[81] MacCallum RC Browne MW Sugawara HM. Power analysis and determination of sample size for covariance structure modeling. Psychol Methods. (1996) 1:130–49. 10.1037/1082-989X.1.2.130

[82] Rigdon EE. CFI versus RMSEA: a comparison of two fit indexes for structural equation modeling. Struct Equ Modeling. (1996) 3:369–79. 10.1080/10705519609540052

[83] Tucker LR Lewis C. A reliability coefficient for maximum likelihood factor analysis. Psychometrika. (1973) 38:1–10. 10.1007/BF02291170

[84] Hooper D Coughlan J Mullen MR. Structural equation modelling: guidelines for determining model fit. Electron J Bus Res Methods. (2008) 6:53–60. 26547545

[85] Browne MW Cudeck R. Alternative ways of assessing model fit. Sociol Methods Res. (1992) 21:230–58. 10.1177/0049124192021002005

[86] Satorra A. Scaled and adjusted restricted tests in multi sample analysis of moment structures. In: Heijmans RDH Pollock DSG Satorra A editors. Department of Economics and Business, Universitat Pompeu Fabra. Boston, MA: Springer (2000). p. 233–247.

[87] Byrne BM Stewart SM. The MACS approach to testing for multigroup invariance of a second-order structure: a walk through the process. Struct Equ Modeling. (2006) 13:287–321. 10.1207/s15328007sem1302_7

[88] Podsakoff PM MacKenzie SB Lee JY Podsakoff NP. Common method biases in behavioral research: a critical review of the literature and recommended remedies. J Appl Psychol. (2003) 88:879–903. 10.1037/0021-9010.88.5.879 14516251

[89] Little TD Cunningham WA Shahar G Widaman KF. To parcel or not to parcel: exploring the question, weighing the merits. Struct Equ Modeling. (2002) 9:151–73. 10.1207/S15328007SEM0902_1

[90] Ko H Cho C-H Roberts MS. Internet uses and gratifications: a structural equation model of interactive advertising. J Advert. (2005) 34:57–70. 10.1080/00913367.2005.10639191

[91] Veldhoven DT Roozen H Vingerhoets A. The association between reward sensitivity and activity engagement: the influence of delay discounting and anhedonia. Alcohol Alcohol. (2020) 55:215–24. 10.1093/alcalc/agz105 31998950 PMC7082492

[92] Masuyama A Kubo T Shinkawa H Sugawara D. The roles of trait and process resilience in relation of BIS/BAS and depressive symptoms among adolescents. PeerJ. (2022) 10:e13687. 10.7717/peerj.13687 35811812 PMC9266581

[93] Hundt NE Mitchell JT Kimbrel NA Nelson-Gray RO. The effect of behavioral inhibition and approach on normal social functioning. Individ Dif Res. (2010) 8:246–56.

[94] Li Q Wang Y Yang Z Dai W Zheng Y Sun Y. Dysfunctional cognitive control and reward processing in adolescents with Internet gaming disorder. Psychophysiology. (2020) 57:e13469. 10.1111/psyp.13469 31456249

[95] Karimpour-Vazifehkhorani A Bakhshipour Rudsari A Rezvanizadeh A Kehtary-Harzang L Hasanzadeh K. Behavioral activation therapy on reward seeking behaviors in depressed people: an experimental study. J Caring Sci. (2020) 9:195–202. 10.34172/jcs.2020.030 33409163 PMC7770387

[96] Holaway RM Heimberg RG Coles ME. A comparison of intolerance of uncertainty in analogue obsessive-compulsive disorder and generalized anxiety disorder. J Anxiety Disord. (2006) 20:158–74. 10.1016/j.janxdis.2005.01.002 16464702

[97] Helbig-Lang S Petermann F. Tolerate or eliminate? A systematic review on the effects of safety behavior across anxiety disorders. Clin Psychol Sci Pract. (2010) 17:218–33. 10.1111/j.1468-2850.2010.01213.x

[98] Wu D Yang T Hall DL Jiao G Huang L Jiao C. COVID-19 uncertainty and sleep: the roles of perceived stress and intolerance of uncertainty during the early stage of the COVID-19 outbreak. BMC Psychiatry. (2021) 21:306. 10.1186/s12888-021-03310-2 34126958 PMC8200549

[99] Muraven M Baumeister RF. Self-regulation and depletion of limited resources: does self-control resemble a muscle? Psychol Bull. (2000) 126:247–59. 10.1037/0033-2909.126.2.247 10748642

[100] Ren J Hu L Zhang H Huang Z. Implicit positive emotion counteracts ego depletion. Soc Behav Pers. (2010) 38:919–28. 10.2224/sbp.2010.38.7.919

[101] Tang Y-Y Posner MI Rothbart MK Volkow ND. Circuitry of self-control and its role in reducing addiction. Trends Cogn Sci. (2015) 19:439–44. 10.1016/j.tics.2015.06.007 26235449

[102] Stein E Witkiewitz K. Trait self-control predicts drinking patterns during treatment for alcohol use disorder and recovery up to three years following treatment. Addict Behav. (2019) 99:106083. 10.1016/j.addbeh.2019.106083 31430618 PMC6791760

[103] Hameed I Irfan BZ. Social media self-control failure leading to antisocial aggressive behavior. Hum Behav Emerg Tech. (2020) 3:296–303. 10.1002/hbe2.226

[104] Watson SJ Milfont T. A short-term longitudinal examination of the associations between self-control, delay of gratification and temporal considerations. Pers Individ Dif. (2017) 106:57–60. 10.1016/j.paid.2016.10.023

[105] Kim J Hong H Lee J Hyun M-H. Effects of time perspective and self-control on procrastination and Internet addiction. J Behav Addict. (2017) 6:229–36. 10.1556/2006.6.2017.017 28494615 PMC5520116

[106] Hannum E Kong P Zhang Y. Family sources of educational gender inequality in rural China: a critical assessment. Int J Educ Dev. (2009) 29:474–86. 10.1016/j.ijedudev.2009.04.007 20161037 PMC2753976

[107] Nolen-Hoeksema S. Emotion regulation and psychopathology: the role of gender. Annu Rev Clin Psychol. (2012) 8:161–87. 10.1146/annurev-clinpsy-032511-143109 22035243

[108] Feng Y Meng D Guo J Zhao Y Ma X Zhu L. Bedtime procrastination in the relationship between self-control and depressive symptoms in medical students: from the perspective of sex differences. Sleep Med. (2022) 95:84–90. 10.1016/j.sleep.2022.04.022 35569330

[109] Chen B Liu F Ding S Ying X Wang L Wen Y. Gender differences in factors associated with smartphone addiction: a cross-sectional study among medical college students. BMC Psychiatry. (2017) 17:341. 10.1186/s12888-017-1503-z 29017482 PMC5634822

[110] Wu Q Chen T Zhong N Bao J Zhao Y Du J. Changes of internet behavior of adolescents across the period of COVID-19 pandemic in China. Psychol Health Med. (2022) 4:1–11. 10.1080/13548506.2021.2019809 34983262

[111] Lin X Gu JY Guo WJ Meng YJ Wang HY Li XJ. The gender-sensitive social risk factors for internet addiction in college undergraduate students. Psychiatry Investig. (2021) 18:636–44. 10.30773/pi.2020.0277 34340274 PMC8328835

[112] Zhang J Bai Z Wei J Yang M Fu G. The status quo of college students' online shopping addiction and its coping strategies. Int J Psychol. (2019) 11:88–93. 10.5539/ijps.v11n2p88

[113] Niedermoser DW Petitjean S Schweinfurth N Wirz L Ankli V Schilling H. Shopping addiction: a brief review. PRI. (2021) 6:199–207. 10.1037/pri0000152

[114] Taylor SE Stanton AL. Coping resources, coping processes, and mental health. Annu Rev Clin Psychol. (2007) 3:377–401. 10.1146/annurev.clinpsy.3.022806.091520 17716061

[115] Canby NK Cameron IM Calhoun AT Buchanan GM. A brief mindfulness intervention for healthy college students and its effects on psychological distress, self-control, meta-mood, and subjective vitality. Mindfulness. (2015) 6:1071–81. 10.1007/s12671-014-0356-5

[116] Smith T Panfil K Bailey C Kirkpatrick K. Cognitive and behavioral training interventions to promote self-control. J Exp Psychol Anim Learn Cogn. (2019) 45:259–79. 10.1037/xan0000208 31070430 PMC6716382

[117] Lopez-Fernandez O. Generalised versus specific internet use-related addiction problems: a mixed methods study on internet, gaming, and social networking behaviours. Int J Environ Res Public Health. (2018) 15:2913. 10.3390/ijerph15122913 30572652 PMC6313434

[118] Su W Han X Yu H Wu Y Potenza MN. Do men become addicted to internet gaming and women to social media? A meta-analysis examining gender-related differences in specific internet addiction. Comput Human Behav. (2020) 113:106480. 10.1016/j.chb.2020.106480

[119] Giacolini T Conversi D Alcaro A. The brain emotional systems in addictions: from attachment to dominance/submission systems. Front Hum Neurosci. (2020) 14:609467. 10.3389/fnhum.2020.609467 33519403 PMC7843379

[120] Alcaro A Brennan A Conversi D. The SEEKING drive and its fixation: a neuro-psycho-evolutionary approach to the pathology of addiction. Front Hum Neurosci. (2021) 15:635932. 10.3389/fnhum.2021.635932 34475816 PMC8406748

[121] Vondráčková P Gabrhelík R. Prevention of Internet addiction: a systematic review. J Behav Addict. (2016) 5:568–79. 10.1556/2006.5.2016.085 27998173 PMC5370363

